# Pharmacists and fastidiousness improve compliance with guidelines for stress ulcer prophylaxis

**DOI:** 10.1186/cc11123

**Published:** 2012-03-20

**Authors:** S Sanders, KC Shelley, AJ Marsh

**Affiliations:** 1 Frenchay Hospital, Bristol, UK

## Introduction

This audit assessed compliance with guidelines for the use of stress ulcer prophylaxis (SUP) in our mixed general/neurosurgical ICU. These patients are at increased risk of gastrointestinal bleeding with clinically important bleeding occurring in about 3.5% of patients ventilated for 48 hours or more [1]. SUP guidelines: all patients at risk of stress ulceration (coagulopathy/IPPV >48 hours/nasogastric (n.g.) feed not absorbed) or already on ant acids should receive ranitidine, enterally where possible. Exceptions are patients on a proton pump inhibitor (PPI) prior to ICU admission. PPIs should continue enterally if possible as lanzoprazole, or as omeprazole i.v.

## Methods

Data were collected from May to August 2010 (Period 1). Results from this were discussed and the following interventions adopted prior to further data collection (Period 2: August to November 2011): prescription of SUP in all ventilated patients on admission to the ICU; discontinuation of SUP after 48 hours if n.g. feeding tolerated; documented daily review of SUP including consideration of discontinuation, drug, route and dose used; and the presence of the ICU pharmacist on ward rounds, briefed specifically to prompt correct SUP use.

## Results

Period 1 (*n *= 86) revealed excess use of SUP, excess use of PPIs when ranitidine was indicated, unnecessary i.v. administration and failure to discontinue prophylaxis appropriately. Period 2 (*n *= 71) demonstrated: no fall in SUP use in those with indications (93% vs. 97%, *P *= 0.65); increased prescription accuracy in terms of drug, dose and administration route (40% vs. 84%, *P *= 0.0001); no increased unindicated SUP use; and reduction in inappropriate i.v. administration (23.1% vs. 0%, *P *= 0.0024).

## Conclusion

Emphasis on the guidelines for SUP to all members of the team, especially the pharmacist, improves compliance. Inclusion in SUP prescriptions of the intended discontinuation date may further reduce excessive duration of treatment. Re-audit will occur after implementation of new guidelines which acknowledge the diminishing benefit from SUP and the not-insignificant risks associated with its use.

## References

[B1] Cook DJ Crit Care 2001 5 368 375 10.1186/cc1071 11737927 PMC83859

